# Dr. James Johnson’s Portraits of the Medical Lecturers of London

**Published:** 1842-11-26

**Authors:** 


*Dr. James Johnson's Portraits of the Medical Lecturers of London.*— The uninitiated might suppose that the lecturers, as a class, were fat monopolists, gorged with fees, and accumulating fortunes. To those who know *the facts* this is positively laughable. Such are the expenses, that some get *nothing*, many get *next to nothing*, most get *very* little, and *very few* receive what can be considered an ample recompense. As for the bulk of the lecturers, one has only to *look* at them to laugh at the idea of their being rich,— *lean, hungry-looking chaps*, with high cheek-bones, *white* complexions, and somewhat *rusty* coats.—*Ibid, from Med. Chir. Rev.*, October, 1842.

---

*Statue of Bichat.*—An iron statue in memory of this distinguished physiologist is about to be erected in one of the squares of Bourg. The work is entrusted to M. David, of Angers.—*London Lancet.*

